# Percutaneous Embolization of Congenital Portosystemic Venous Fistula in an Infant with Down Syndrome

**DOI:** 10.1155/2013/127023

**Published:** 2013-09-19

**Authors:** Pattaraporn Tanya Chun, Terrence Chun, Matthew Files, Nghia Vo, Ryan M. McAdams

**Affiliations:** ^1^Department of Pediatrics, Division of Neonatology, University of Washington, Box 356320, Seattle, WA 98195-6320, USA; ^2^Department of Pediatrics, Division of Cardiology, University of Washington, Seattle, WA 98195-6320, USA; ^3^Department of Radiology, University of Washington, Seattle, WA 98195-6320, USA; ^4^Department of Radiology, Medical College of Wisconsin and Children's Hospital of Wisconsin, Milwaukee, WI 53201-1997, USA

## Abstract

Congenital intrahepatic portosystemic venous shunts are rare vascular malformations often associated with severe complications. We describe a term male infant with Down syndrome with high output heart failure secondary to a congenital arterial to portal venous fistula that was diagnosed by Doppler ultrasound. Percutaneous embolizations of the left hepatic vein, portal vein, and communicating fistulas were performed without complications, resulting in clinical improvement. A subsequent hepatic ultrasound demonstrated resolution of the pathologic fistulous communication and shunting effects.

## 1. Introduction

Congenital portosystemic shunts (CPSS) have been classified into extrahepatic and intrahepatic based on the location of the shunts [1]. These vascular malformations can be detected by ultrasonography (US), computed tomography (CT) scan, and magnetic resonance (MR) angiography. Complications of CPSS may manifest in early infancy or later in life, including neonatal hyperammonemia, cholestasis, hypergalactosemia, liver tumors (benign or malignant), portosystemic encephalopathy, pulmonary arterial hypertension, right heart failure, and hepatopulmonary syndrome [2]. We report the case of an infant with Down syndrome presenting with high output cardiac failure discovered to be due to intrahepatic CPSS who was successfully treated with a percutaneous embolization procedure. 

## 2. Case Report

A term, 2662 grams (6.6 percentile), small for gestational age male infant born by spontaneous vaginal delivery at an outside institution, developed hypoxic respiratory failure requiring continuous positive pressure ventilation. His prenatal history was significant for intrauterine growth restriction, trisomy 21 (47, XY, +21) diagnosed by amniocentesis, and a reportedly normal fetal echocardiogram. On day of age 2, he was noted to have an increasing pre- and postductal saturations differential, requiring a FiO_2_ of 0.8 by nasal cannula to keep his oxygen saturations 95%. A limited echocardiogram was concerning for persistent pulmonary hypertension (PPHN), demonstrating exclusive right to left ductal shunting with a dilated right atrium and right ventricle, so the infant was placed on inhaled nitric oxide at 20 parts per million, which increased his pre- and post-ductal saturations. Additionally, a dilated vascular hepatic structure with continuous flow was noted on ultrasound. Due to persistent respiratory distress, the infant was intubated, placed on mechanical ventilation, and transferred to our institution for further evaluation of his suspected liver vascular malformation.

Physical examination was significant for Down syndrome facies and hypotonia. Cardiovascular exam revealed an accentuated precordial impulse and a faint continuous murmur audible at the left axilla. Pulses were diminished. The abdominal examination was normal without hepatomegaly. No bruits were appreciable over the liver. Laboratory studies were significant for thrombocytopenia (platelet count 1 × 10^9^/L), normal serum liver transaminases, and an arterial blood gases without metabolic acidosis. A 12-lead electrocardiogram was also normal for his age. A detailed echocardiogram confirmed right to left shunting and additionally demonstrated a large collateral arterial vessel from the left subclavian artery that coursed anteriorly over the heart before entering the liver vascular malformation. The descending aorta Doppler signal also raised suspicion for additional arterial supply to the vascular malformation. 

**Figure 1:**
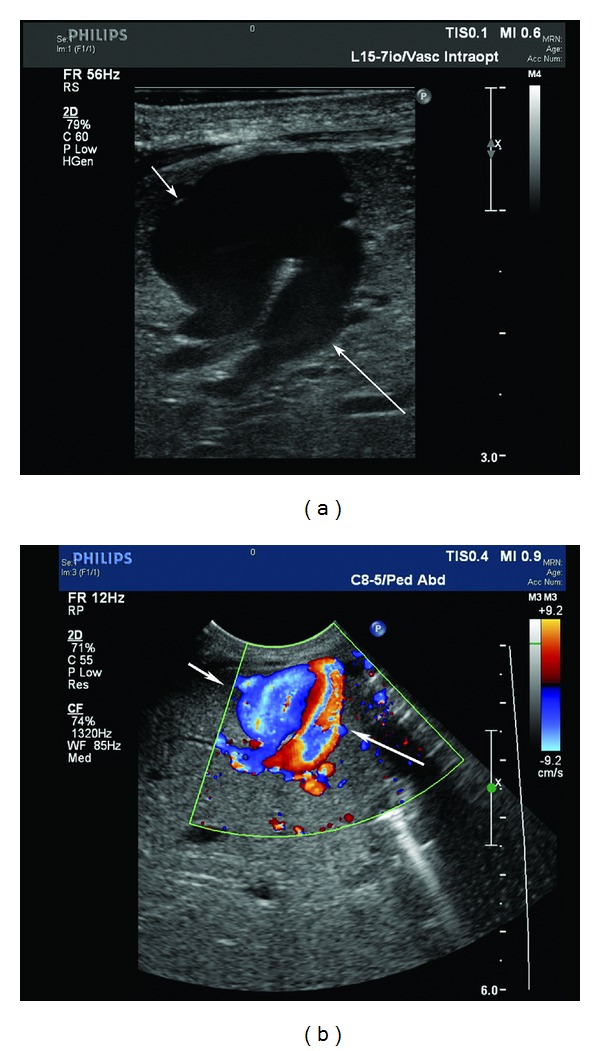
(a) Gray-scale ultrasound of the fistulous communication between the left portal vein (long arrow) and left hepatic vein (short arrow). (b) Color Doppler ultrasound image of the junctional fistulous communication point demonstrating inflow in the left portal vein (long arrow) and outflow in the left hepatic vein (short arrow).

**Figure 2:**
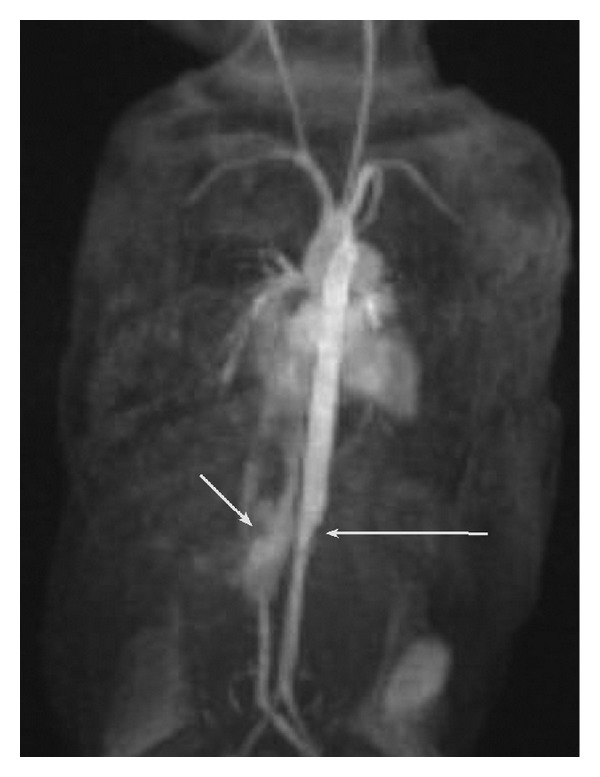
Coronal postcontrast twist sequence (time resolved gadolinium enhanced MRI) with multiphase following intravenous injection of gadopentetate dimeglumine. Arterial phase evaluation of the abdomen demonstrated a definite change in caliber of the abdominal aorta at the level of the midliver with narrowing of its subdiaphragmatic portion (long arrow). Additionally, between the IVC and the aorta, in the region of the left lobe of the liver, a vascular structure fills the left lobe of the liver (short arrow), communicating with a large left hepatic vein draining into the right atrium.

An abdominal ultrasound (Figure 1), MR angiogram (Figure 2), and CT with contrast of the chest and abdomen were obtained to better characterize the vascular lesion. These studies demonstrated an abnormal vascular communication in the left lobe of his liver between the left portal vein and an enlarged left hepatic vein. The Doppler pattern showed an arterialized waveform suggestive of an extraneous arterial supply to the venous malformation, which was supported by the MR findings demonstrating a change in caliber of the subdiaphragmatic aorta, enlarged celiac artery, and enlarged left internal mammary artery supplying parts of the lesion in the left lobe of the liver. The low systemic vascular resistance of the arterial-venous fistula was suspected to be responsible for the large cardiac shunt at the ductus arteriosus and resulting high output heart failure. 

**Figure 3:**
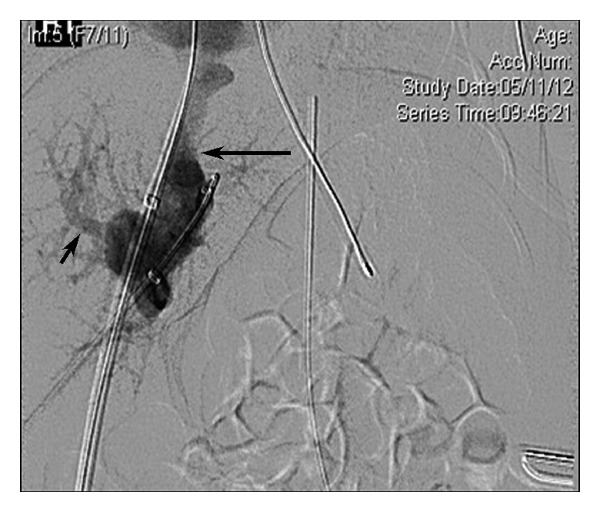
Percutaneous venogram demonstrating opacification of the right portal vasculature (short arrow) and the left hepatic vein (long arrow) from a manual injection following percutaneous access at the junctional fistulous communication point between the left portal vein and left hepatic vein.

At 6 days of age, to improve pulmonary artery blood flow and balance the infant's circulation, he was taken to the interventional radiology suite for percutaneous embolization of this vascular malformation (Figure 3). A terminal intrahepatic arterial structure that directly communicated with the portal vein and hepatic vein fistula was visualized by ultrasound. An arterialized waveform was seen in the fistulous communication itself using Doppler. Under ultrasound guidance, access was gained to the fistulous connection and sheaths were placed over a wire in the left hepatic vein and the left portal vein. Amplatzer vascular plugs (St. Jude Medical, St. Paul, MN), 8 mm and 12 mm, were used to occlude the left hepatic vein outflow, and an 8 mm Amplatzer vascular plug was placed in the left portal vein. Coils were packed between the Amplatzer plugs in the communicating fistulous junctional zone. The arterial vessels feeding into the fistula no longer directly communicated and had no outflow source after occlusion and closure of the fistulous communication across the segment. As a result, no arterial side intervention was necessary. 

Following the procedure, the inhaled nitric oxide was weaned off and the infant was easily extubated to room air. Subsequent follow-up Doppler US imaging showed no further evidence of residual arterio-portal-venous shunting with resolution of the multiple previously identified vascular channels. The remaining underlying left hepatic parenchyma had an otherwise normal sonographic appearance. A repeat echocardiogram showed that the ductal level shunting was exclusively left-to-right without additional signs of pulmonary hypertension. The infant was discharged home at 12 days of age. By two months of age, his cardiac dimensions had entirely normalized with only a trivial residual left-to-right ductal shunting. A hepatic ultrasound at 4 months of age demonstrated no evidence of recurrent arterio-portal-venous shunting. He had continued to achieve age appropriate developmental milestones.

## 3. Discussion

CPSS are rare vascular malformations. The clinical presentation of CPSS malformations varies from asymptomatic to symptomatic and can be associated with serious complications, including neonatal cholestasis, hyperammonemia, hypergalactosemia, and portosystemic encephalopathy, none of which was manifested by this patient [3]. Decreased liver perfusion due to vascular shunting can lead to growth restriction [4] and portal hypertension [5]. 

Postnatal imaging tools to diagnose CPSS include US, MRI/MRA, and CT. We found Doppler US, which demonstrated an arterialized waveform, to be most useful in determining that an arterial-to-venous shunt was present. MRI/MRA, while confirming the presence of an arterial-to-venous shunt, was no more informative than US, whereas CT did not demonstrate an arterial component of the CPSS. In most cases, Doppler US appears to be the favored imaging modality for diagnosing CPSS since it does not require movement of the patient to the scanner, expose the patient to radiation, or require sedation of the patient and is relatively inexpensive. 

The association between chromosomal abnormalities, including Down syndrome, and CPSS has been reported [6]. Although it has been postulated that the risk of congenital vascular malformations is decreased with Down syndrome due to the presence of vascular epithelial growth factor inhibitors on chromosome 21 [6], these malformations can occur in Down syndrome patients. In the present case, an anomalous portal venous communication was maintained by an intrahepatic venous fistula between the left portal vein and left hepatic vein and was additionally supplied by systemic arterial collateral vessels. This abnormal arteriovenous shunt resulted in an abnormally low systemic vascular resistance relative to normal postnatal pulmonary vascular resistance, perpetuating the fetal circulation of a right-to-left ductal level shunt. As a result, the patient's initial clinical presentation was consistent with PPHN, a syndrome characterized by marked pulmonary hypertension that causes hypoxemia and right-to-left extrapulmonary shunting of blood. Since neonates with Down syndrome have a known increased incidence of idiopathic PPHN, the PPHN in this case did not immediately suggest an abnormality such as CPSS. This finding supports obtaining echocardiograms in infants with suspected PPHN to identify cardiac and less common abnormalities, such as the left subclavian artery collateral vessel connecting to the liver that was seen in our case.

Spontaneous portosystemic shunt closure can occur within the first 2 years of age [1]. Asymptomatic shunts may remain until childhood. However, in the setting of severe pulmonary artery hypertension or right heart failure, closure of the shunts either by endovascular or surgical approach should be considered. Franchi-Abella et al. have proposed guidelines for the closure of CPSS in children [2]. 

Our patient was at risk for high output cardiac failure due to increased venous return caused by the low resistance hepatic circulation. Percutaneous management is less invasive than an open surgical approach and is therefore the preferred treatment for CPSS malformations. Interventional radiology successfully performed percutaneous embolization of the fistulous communication between the infant's left hepatic and portal veins. Embolization of both the hepatic venous outflow and the portal venous connections was performed to prevent possible proliferation due to the additional arterial supply of the vascular malformation. Acute portal hypertension has been reported to be associated with portosystemic shunt closure [7]. This complication may be detected by measuring the portal pressure prior to shunt closure (occlusion test). It has been suggested that a single step endovascular shunt closure should not be done for portal pressures greater than 32 mmHg [8]. In cases of elevated portal pressures, a two-step approach with surgical banding of the shunt followed by endovascular closure is preferred [2]. Patients who do not respond to these interventions and have persistent portal hypertension may require a future liver transplant. 

## 4. Conclusion

Although rare, infants with Down syndrome presenting with PPHN may have underlying CPSS. Doppler ultrasonography is useful in diagnosing CPSS in early infancy. Percutaneous endovascular closure of the shunts has been reported as the standard management of these malformations [2]. Subsequent patient followup with imaging and measuring serum transaminases is needed due to an increase risk of portal hypertension after shunt closure.
